# Women empowerment and health insurance utilisation in Rwanda: a nationwide cross-sectional survey

**DOI:** 10.1186/s12905-022-01976-8

**Published:** 2022-09-16

**Authors:** Joseph Kawuki, Ghislaine Gatasi, Quraish Sserwanja

**Affiliations:** 1 Centre for Health Behaviours Research, Jockey Club School of Public Health and Primary Care, The Chinese University of Hong Kong, Sha Tin, Hong Kong, SAR China; 2 Key Laboratory of Environmental Medicine Engineering, School of Public Health, Southeast University, Nanjing, 210009 Jiangsu Province China; 3 Programmes Department, GOAL, Khartoum, Sudan

**Keywords:** Health insurance, Utilisation, Coverage, Women empowerment, Rwanda

## Abstract

**Background:**

Health insurance coverage is one of the several measures being implemented to reduce the inequity in access to quality health services among vulnerable groups. Although women’s empowerment has been viewed as a cost-effective strategy for the reduction of maternal and child morbidity and mortality, as it enables women to tackle the barriers to accessing healthcare, its association with health insurance usage has been barely investigated. Our study aims at examining the prevalence of health insurance utilisation and its association with women empowerment as well as other socio-demographic factors among Rwandan women.

**Methods:**

We used Rwanda Demographic and Health Survey (RDHS) 2020 data of 14,634 women aged 15–49 years, who were selected using multistage sampling. Health insurance utilisation, the outcome variable was a binary response (yes/no), while women empowerment was assessed by four composite indicators; exposure to mass media, decision making, economic and sexual empowerment. We conducted multivariable logistic regression to explore its association with socio-demographic factors, using SPSS (version 25).

**Results:**

Out of the 14,634 women, 12,095 (82.6%) (95% CI 82.0–83.2) had health insurance, and the majority (77.2%) were covered by mutual/community organization insurance. Women empowerment indicators had a negative association with health insurance utilisation; low (AOR = 0.85, 95% CI 0.73–0.98) and high (AOR = 0.66, 95% CI 0.52–0.85) exposure to mass media, high decision making (AOR = 0.78, 95% CI 0.68–0.91) and high economic empowerment (AOR = 0.63, 95% CI 0.51–0.78). Other socio-demographic factors found significant include; educational level, wealth index, and household size which had a negative association, but residence and region with a positive association.

**Conclusions:**

A high proportion of Rwandan women had health insurance, but it was negatively associated with women’s empowerment. Therefore, tailoring mass-media material considering the specific knowledge gaps to addressing misinformation, as well as addressing regional imbalance by improving women’s access to health facilities/services are key in increasing coverage of health insurance among women in Rwanda.

## Background

Achieving Universal health coverage (UHC) is one of the sustainable development goals (SDGs) targets to be achieved by 2030, and it has been shown to lead to progress toward other health-related targets, and the other goals [1]. UHC ensures that people and communities can get the required health services without suffering financial hardships [1, 2]. Before the COVID-19 pandemic, over 927 million people (12.7%) globally, made out-of-pocket health-care payments which were above 10% of their household budgets, pushing over 90 million people below the extreme poverty line [3].

Besides protecting people against health expenditure-induced poverty, health insurance also ensures quality and comprehensive access to all aspects of healthcare from health promotion to prevention, treatment, rehabilitation and palliative care [1–3]. Despite access to quality skilled health care being one of the main factors associated with a reduction in maternal morbidity and mortality, inequity in access to care is more common among women, especially in developing countries [4–6]. This is attributed mainly to their limited access to economic resources, lower education levels, and employment in less paying and informal jobs, compared to their male counterparts, who in most cases tend to be in more privileged socio-economic hierarchies [4, 7, 8]. In 2020, 35% of nations globally reported interruptions in access to nutrition, maternal and health services which led to 228,000 additional child deaths [3]. Nonetheless, there is evident inequity in access to healthcare, particularly in developing countries compared to developed ones where access to healthcare is/tends to be universal [9].

Analyzing demographic and health Surveys in 36 Sub-Saharan African (SSA) countries, Tessema et al. showed healthcare access among women of reproductive age in SSA to be at only 42.6% [10], while Minyihun et al. analysed demographic and health Surveys in 12 East African countries showed access to care at 42.9% [4]. Although Rwanda achieved the Millennium Development Goals (MDG) 4 and 5 [11, 12] and registered great improvement in the maternal mortality ratio (MMR) between 2005, 2010 and 2015, a slow pattern has been registered between 2015 and 2020; from 210 deaths per 100,000 live births to just 203 deaths per 100,000 live births [13, 14]. Furthermore, access to some indicators of maternal health such as the utilization of at least four antenatal care contacts is still very low at 47% [14], which is far below the global targets, since the World Health Organization (WHO) modified the minimum number of ANC visits from four to at least eight contacts in 2016 [15–17].

Several factors have been documented to affect access and utilization of maternal healthcare. Among the commonest and crucial factors are the direct and indirect costs incurred by women [18, 19]. Women from poor households experience the worst effects of out-of-pocket health expenditure [18, 20]. Several measures and policies are being implemented in Africa to improve access to and coverage of health services. One of these is national health insurance schemes [2, 21]. These schemes aim at increasing access to quality healthcare focusing mainly on the poor and vulnerable population and have been shown to be positively associated with utilization and access to maternal healthcare [2, 22]. Rwanda’s Mutuelles de santé—known in English as the community-based health insurance (CBHI) program was initiated in 2004, with its coordination being handled at the district level and enrollment support from village mobilization committees [21, 23]. Besides CBHI, several other insurance schemes exist in Rwanda such as the formal sector Rwandaise d’Assurance Maladie (RAMA)- known as Rwandan health insurance, military medical insurance (MMI), as well as other privately purchased schemes [23]. However, most of the population (over 80%) utilizes CBHI—it being the mandated option for uninsured citizens, especially from rural settings and those in the informal sector [24, 25]. Although it was initiated to focus on the poor, CBHI utilization has been highly skewed towards the rich [21]. The CBHI premiums are income-sensitive and remain a barrier for the poor to enrol on the scheme [21, 26].

Globally, the definition of women empowerment has varied substantially and its measurement is still controversial; and therefore commonly inferred by indicators, such as decision-making, access to information, financial independence, or mobility freedom and some other variables such as attitude towards gender-based violence and support from family, amongst others [27]. Women empowerment has been viewed as a cost-effective strategy for the reduction of maternal and child morbidity and mortality by improving decision-making ability and access to economic resources, hence being in a better position to tackle the barriers to accessing healthcare [5, 28]. Empowered women have been shown to have better health-seeking behaviour and decision-making [29, 30]. Moreover, with increased access to economic resources, women’s empowerment would ease and increase subscription to health insurance schemes, translating into increased access to healthcare. Nonetheless, Rwanda ranks among the best countries in women empowerment with women scoring higher than men in some indicators such as literacy (73.2% vs. 69.4%), seats held in the national parliament (61.3% vs. 38.7%) and out-of-school children (5.2% vs. 6.3%) [31]. Most of the available studies have explored factors associated with health insurance usage among women in other settings other than Rwanda [32–37], with none focusing on its association with women empowerment. In order to ensure an increase in access to healthcare by women, evidence is needed to assess the association between women’s empowerment indices and enrollment into health insurance schemes. Therefore, our study aims at examining the association between women’s empowerment, including other socio-demographics and the utilization of health insurance in Rwanda.

## Methods

### Study sampling and participants

We used the 2019–20 Rwanda Demographic Survey (RDHS) for this analysis, which was a cross-sectional study and employed a two-stage sample design, with the first stage involving cluster selection consisting of enumeration areas (EAs) [14]. The second stage involved systematic sampling of households in all the selected EAs leading to a total of 13,005 households [14]. In particular, the data used in this analysis were from the household and the woman’s questionnaires.

During this survey, the data collection period was from November 2019 to July 2020, taking longer than expected due to the COVID-19 pandemic restrictions [14]. Women aged 15–49 years who were either permanent residents of the selected households or visitors who had stayed in the household the night before the survey were eligible to be interviewed. Out of the total 13,005 households that were selected for the survey, 12,951 were occupied and 12,949 were successfully interviewed leading to a 99.9% response rate [14]. This analysis included all women interviewed during the survey, and of the selected households, 14,675 women aged 15–49 were eligible to be interviewed but 14,634 women were successfully interviewed leading to a 99.7% response rate [14].

### Variables

#### Dependent variables

The study outcome variable was the usage of health insurance. This was defined if a respondent had any type of insurance that covers the whole or a part of the risk incurred from medical expenses, and was a binary variable directly coded yes or no [14].

### Explanatory variables

#### Measures of women’s empowerment

Four indices were created to measure the empowerment of women: exposure to media, decision making, economic empowerment, and sexual empowerment. Women’s empowerment indices were measured as composite scores [5, 27].

Exposure to media was considered as the women’s ability to have the opportunity to read a newspaper or a magazine, listen to the radio and watch TV. Responses were re-coded (1 if the woman was exposed to newspapers, radio or TV and 0 if the woman was not). We then created an index, by adding all the scores for each woman, with the total score ranging from 0 to 3, after which we finally categorized the scores into four groups [5]. A total score of 0 meant no access to any of the three media, while scores of 1(low), 2(medium) and 3(high) implied exposure to one, two, and three media channels respectively [5, 27].

Decision-making included women’s ability to be involved in making decisions regarding; their own health; large household purchases; visits to their family and control over family earnings [5]. We re-coded the responses to have two categories (1 = woman involved in decision making alone or with a partner, 0 = woman not involved in decision making). We then added all the scores to form an index score ranging from 0 to 4, and we finally categorized the score into four groups. The highest score was four which meant that the woman was involved in the decision-making for the four used indicators. Medium decision-making ability meant that women were involved in 2 or 3 indicators, low decision making meant that the woman was involved in only one indicator and no decision making implied that the woman was not involved in any decision making [5, 27, 28].

Economic empowerment entailed women’s owning of a house, land and the type of earning from her work [5, 27]. We re-coded the three indicators as 1-if the women owned a house or land (either alone or jointly with a partner) or received cash payment for their work and 0-if didn’t own a house, land or cash payment for work. An index was then created by summing the scores for each woman, with a total score ranging from 0 to 3, after which we categorized the score into four groups. The highest score of 3 implied that the woman owned a house, land, and earned cash for her work, while scores of 2, 1 and 0 meant medium, low and no economic empowerment, respectively.

Sexual empowerment referred to the women’s ability to refuse sex and ask a partner to use condoms [5, 38]. Responses were coded (1 if the woman could refuse sex or ask for a condom and 0 if the woman could not) and sexually empowered women were those who were able to refuse sex or ask their partners to use condoms. We then created an index by adding the scores for each woman with a total score ranging from 0 to 2, after which we categorized the score into three groups. The highest score of 2 implied high sexual empowerment, while scores of 1 and 0 respectively meant low and no sexual empowerment.

Decision-making and sexual empowerment had about 7233 missing responses, while economic empowerment had about 3908 missing values, and this was because some of these questions were asked during the domestic violence survey sessions, yet not all women in the RDHS were included in the domestic violence module of the survey. These missing observations were assumed to be zero [5], thus we risked overestimating low subcategories of these composite indices/variables. To ensure that this doesn’t affect our findings, we conducted a sensitivity analysis by considering only women sampled in the domestic violence model and excluded those with missing responses. However, this showed no significant difference from the original analysis and more details are included in the sensitivity analysis section of the results. Moreover, for background characteristics, we provided frequencies of these variables considering only women with valid responses.

### Other socio-demographic variables

We included possible determinants of health insurance utilisation based on available literature and data [32–37]. Ten (10) variables were considered and of these, two were community-level factors that included; place of residence and region of residence. Three household-level factors included; household size, sex of household head and wealth index. Wealth index was calculated by RDHS from information on household asset ownership using Principal Component Analysis [14]. Five individual-level factors were also considered in the analysis, including; age, educational level, working status, marital status, and religion. None of the included variables was a potential mediator of the the main relationship of interest (that is, health insurance and women empowerment).

### Statistical analysis

We applied the DHS sample weights to account for the unequal probability sampling in different strata and ensure the representativeness of the study results [39, 40]. We used Statistical Package for Social Sciences (SPSS) software (version 25.0) with a complex samples package, incorporating the following variables in the analysis plan to account for the multistage sample design inherent in the RDHS dataset: individual sample weight, sample strata for sampling errors/design, and cluster number [14, 39]. Initially, we did descriptive statistics for both dependent and independent variables. Frequencies and proportions/percentages for categorical dependent and independent variables have been presented. Afterwards, bivariable logistic regression was done to assess the association of each independent variable (i.e. women empowerment indicators and various socio-demographic factors) with health insurance utilisation and crude odds ratio (COR), 95% confidence interval (CI) and *p*-values are presented. Independent variables found significant at the bivariable level with *p*-values less than 0.25 were then included in the multivariable model, including factors known to be associated with health insurance usage based on previous studies, regardless of their significance. The final model controlled for all the included factors, where we calculated and presented their respective adjusted odds ratios (AOR), 95% CI and *p*-values, at a statistical significance level of 0.05.

Since questions of decision making and sexual empowerment were asked to only women selected for the domestic violence module, we conducted a sensitivity analysis where we considered only women with domestic violence module responses, excluding those with no (missing) such responses. All socio-demographic variables in the model were assessed for multi-collinearity, which was considered present if the variables had a variance inflation factor (VIF) greater than 10 [41]. However, none of the variables had a VIF above 3.

## Results

A total of 14,634 women were included in this analysis (Table 1). The majority were aged 15–34 years (67.1%), had primary or secondary education (86.2%), were working (66.3%), were rural residents (80.1%), and from male-headed households (68.9%) of less than 6 household members (59.3%). Moreover, the majority of the women had low to medium mass media exposure (63.9%), low to medium economic empowerment (67.9%), medium to high decision making (89%) and high sexual empowerment (64.3%), as detailed in Table 1.

**Table 1:** Background characteristics of Rwandan women aged 15–49 years as per the 2020 RDHS

Characteristics	Frequency (%), N = 14,634
*Age*	
45–49	1211 (8.3)
35–44	3560 (24.3)
25–34	4191 (28.6)
15–24	5672 (38.8)
*Education level*	
Tertiary	642 (4.4)
Secondary	4086 (27.9)
Primary	8529 (58.3)
No education	1377 (9.4)
*Working status*	
Working	9702 (66.3)
Not working	4932 (33.7)
*Marital status*	
Married	7401 (50.6)
Unmarried	7233 (49.4)
*Religion*	
Catholic	5364 (36.7)
Protestant	6905 (47.2)
Adventist	1836 (12.5)
Muslim	269 (1.8)
Others	261 (1.8)
*Wealth index*	
Richest	3414 (23.3)
Richer	2966 (20.3)
Middle	2757 (18.8)
Poorer	2756 (18.8)
Poorest	2741 (18.7)
*Residence*	
Urban	2909 (19.9)
Rural	11,725 (80.1)
*Region*	
Kigali	2166 (14.8)
West	3174 (21.7)
East	4003 (27.4)
North	2226 (15.2)
South	3065 (20.9)
*Household size*	
Less than 6	8672 (59.3)
6 and above	5962 (40.7)
*Sex of household head*	
Male	10,081 (68.9)
Female	4553 (31.1)
*Exposure to mass media*	
No exposure	2522 (17.2)
Low	4894 (33.4)
Medium	4460 (30.5)
High	2758 (18.8)
*Economic empowerment*^*a*^	
No	1269 (11.8)
Low	4211 (39.3)
Medium	3072 (28.6)
High	2174 (20.3)
*Decision-making*^*b*^	
No	347 (4.7)
Low	467 (6.3)
Medium	2265 (30.6)
High	4322 (58.4)
*Sexual empowerment*^*b*^	
No	827 (11.2)
Low	1812 (24.5)
High	4762 (64.3)

Missing values, a = 3908, b = 7233

Of the 14,634 women, 12,095 (82.6%) (95% CI 82.0–83.2) were using or had subscribed to health insurance. The majority were covered by mutual/community organization insurance (77.2%), followed by social security coverage (3.6%) and military medical insurance coverage (1.2%), Table 2.

**Table 2:** Types of Health insurance used

Health insurance coverage	Frequency (%), N = 14,634
**Yes**	**12,095 (82.6) (95% CI 82.0–83.2)**
No	2539 (17.4) (95% CI 16.8–18.0)

Insurance type/provider	Frequency (%), N = 14,634
Mutual/community organization	11,292 (77.2)
Social security	531 (3.6)
Military medical insurance	178 (1.2)
Private/commercially purchased	94 (0.6)
Provided by employer	14 (0.1)

Bold value indicate outcome variable

### Factors associated with health insurance utilisation

Results of the bivariable analysis are detailed in Table 3, where all the four measures of women empowerment had significant associations with health insurance usage. Apart from age and residence, all the other selected socio-demographic factors had significant associations with bivariable analysis. In the final logistic regression model, the women empowerment indicators significantly associated with health insurance utilisation were; exposure to mass media, decision making and economic empowerment. Women’s sexual empowerment was not a significant factor. Compared to women with no mass media exposure, those with low (AOR = 0.85, 95% CI 0.73–0.98) and high exposure (AOR = 0.66, 95% CI 0.52–0.85) were 15%, and 34%, respectively, less likely to have health insurance coverage. Compared to their counterparts with no decision-making, women with high decision-making (AOR = 0.78, 95% CI 0.68–0.91) were 22% less likely to use health insurance. Moreover, compared to those with no economic empowerment, women with high economic empowerment (AOR = 0.63, 95% CI 0.51–0.78) were also 37% less likely to use health insurance.

**Table 3:** Factors associated with Health insurance utilisation among women in Rwanda as per the 2020 RDHS

Variable	Crude odds ratio, COR (95% CI)	*p*-value*	Adjusted odds ratio, AOR (95% CI)	*p*-value**
*Empowerment*				
Exposure to mass media		** < 0.001**		**0.001**
No exposure	1		1	
Low	**0.61 (0.53–0.70)**		**0.85 (0.73–0.98)**	
Medium	**0.59 (0.51–0.68)**		0.95 (0.80–1.13)	
High	**0.32 (0.26–0.39)**		**0.66 (0.52–0.85)**	
Decision making		** < 0.001**		**0.001**
No	1		**1**	
Low	**1.30 (0.98–1.69)**		1.20 (0.88–1.59)	
Medium	**0.96 (0.83–1.10)**		0.92 (0.78–1.08)	
High	**0.71 (0.64–0.80)**		**0.78 (0.68–0.91)**	
Economic empowerment		** < 0.001**		** < 0.001**
No	1		1	
Low	**1.36 (1.20–1.53)**		1.13 (0.986–1.29)	
Medium	**1.03 (0.91–1.17)**		0.88 (0.74–1.03)	
High	**0.71 (0.61–0.84)**		**0.63 (0.51–0.78)**	
Sexual empowerment		** < 0.001**		0.108
No	1		1	
Low	0.87 (0.75–1.01)		0.84 (0.66–1.07)	
High	**0.79 (0.71–0.88)**		0.78 (0.62–0.98)	
*Other variables*				
Age		0.432		0.629
15–24	1		1	
25–34	1.03 (0.92–1.16)		1.05 (0.91–1.22)	
35–44	1.09 (0.98–1.22)		1.06 (0.90–1.25)	
45–49	1.03 (0.87–1.21)		0.95 (0.77–1.17)	
Education level		** < 0.001**		** < 0.001**
No education	1		1	
Primary	**0.61 (0.53–0.70)**		**0.75 (0.64–0.87)**	
Secondary	**0.33 (0.28–0.40)**		**0.51 (0.41–0.63)**	
Tertiary	**0.11 (0.07–0.19)**		**0.25 (0.15–0.40)**	
Working status		**0.009**		0.862
Not working	1		1	
Working	**1.15 (1.04–1.28)**		1.01 (0.89–1.16)	
Marital status		**0.004**		0.263
Unmarried	1		1	
Married	**0.87 (0.78–0.95)**		1.19 (0.88–1.62)	
Religion		**0.010**		0.436
Catholic	1		1	
Protestant	**1.26 (1.10–1.44)**		1.05 (0.91–1.2)	
Adventist	1.08 (0.89–1.31)		1.00 (0.82–1.23)	
Muslim	1.10 (0.72–1.68)		1.10 (0.72–1.69)	
Others	**1.70 (1.21–2.40)**		1.42 (0.97–2.09)	
Wealth index		** < 0.001**		** < 0.001**
Poorest	1		1	
Poorer	**0.51 (0.43–0.60)**		**0.52 (0.44–0.62)**	
Middle	**0.38 (0.31–0.47)**		**0.39 (0.32–0.48)**	
Richer	**0.30 (0.24–0.36)**		**0.24 (0.20–0.30)**	
Richest	**0.20 (0.16–0.24)**		**0.13 (0.09–0.17)**	
Residence		0.613		** < 0.001**
Rural	1		1	
Urban	0.95 (0.79–1.15		**1.72 (1.32–2.24)**	
Region		** < 0.001**		** < 0.001**
South	1		1	
North	**0.61 (0.47–0.80)**		**0.68 (0.51–0.90)**	
East	**1.31 (1.05–1.65)**		**1.70 (1.34–2.15)**	
West	**1.27 (1.01–1.58**)		**1.36 (1.08–1.73)**	
Kigali	1.26 (0.98–1.61)		**2.80 (2.10–3.74)**	
Household size		**0.200**		** < 0.001**
6 and above	1		1	
Less than 6	**0.92 (0.81–1.04)**		**0.78 (0.68–0.89)**	
Sex of household head		** < 0.001**		0.196
Female	1		1	
Male	**0.75 (0.67–0.85)**		0.92 (0.80–1.05)	

*RDHS* Rwanda demographic health survey

Bold values indicate significant, * = significant at 0.25, ** = significant at 0.05

Regarding other socio-demographic factors, those found to be significantly associated with health insurance usage included; education level, wealth index, residence, region, and household size. Compared to women with no education, those with primary (AOR = 0.75, 95% CI 0.64–0.87), secondary (AOR = 0.51, 95% CI 0.41–0.63) and tertiary (AOR = 0.25, 95% CI 0.15–0.40) education were 25%, 49% and 75% less likely to use health insurance, respectively. Compared with women of the poorest wealth quintile, those in the poorer (AOR = 0.52, 95% CI 0.44–0.62), middle (AOR = 0.39, 95% CI 0.32–0.48), richer (AOR = 0.24, 95% CI 0.20–0.30), and richest (AOR = 0.13, 95% CI 0.09–0.17) quintiles were 48%, 61%, 76% and 87% less likely to have health insurance coverage, respectively. Moreover, women residing in urban areas (AOR = 1.72, 95% CI 1.32–2.24) were 72% more likely to use health insurance compared to their rural counterparts. Similarly, compared to those in the Southern region, women residing in Kigali (AOR = 2.80, 95% CI 2.10–3.74), Eastern (AOR = 1.70, 95% CI 1.34–2.15), and Western (AOR = 1.36, 95% CI 1.08–1.73) regions were 180%, 70% and 36% more likely to have health insurance, respectively, unlike those in the Northern region (AOR = 0.68, 95% CI 0.51–0.90) whose were 32% less likely to have health insurance. Women from households of less than 6 members (AOR = 0.78, 95% CI 0.68–0.89) were also 22% less likely to have health insurance, compared with those from bigger households.

### Sensitivity analysis considering only women with no missing values

Results of sensitivity analysis are detailed in Table 4. Regarding women empowerment indicators, no big difference was observed in the sensitivity analysis model, and the same factors remained significant. However, regarding other socio-demographic factors, the sex of the household head become significantly associated with health insurance utilisation, no big difference was observed among the other factors, apart from marital status which was not applicable (since only married women responded to the domestic violence module questions). Compared with women from female-headed households, those from male-headed households (AOR = 0.78, 95% CI 0.63–0.96) were 22% less likely to have health insurance.

**Table 4:** Sensitivity analysis considering only women with domestic violence module responses per the 2020 RDHS

Variable	Adjusted odds ratio, AOR (95% CI)	*p*-value**
*Empowerment*		
Exposure to mass media		**< 0.001**
No exposure	1	
Low	**0.73 (0.60–0.89)**	
Medium	0.99 (0.79–1.23)	
High	**0.47 (0.33–0.67)**	
Decision making		**0.007**
No	**1**	
Low	1.01 (0.67–1.51)	
Medium	0.77 (0.55–1.07)	
High	**0.67 (0.48–0.94)**	
Economic empowerment		**< 0.001**
No	1	
Low	1.12 (0.87–1.45)	
Medium	0.85 (0.6–1.12)	
High	**0.61 (0.45–0.83)**	
Sexual empowerment		0.102
No	1	
Low	0.84 (0.65–1.08)	
High	0.77 (0.61–0.98)	
*Other variables*		
Age		0.548
15–24	1	
25–34	1.08 (0.84–1.38)	
35–44	1.13 (0.85–1.50)	
45–49	0.96 (0.68–1.35)	
Education level		**< 0.001**
No education	1	
Primary	**0.74 (0.61–0.91)**	
Secondary	**0.52 (0.38–0.71)**	
Tertiary	**0.29 (0.14–0.59)**	
Working status		0.552
Not working	1	
Working	1.06 (0.87–1.30)	
Marital status		N/A
Unmarried	N/A	
Married	N/A	
Religion		0.868
Catholic	1	
Protestant	1.08 (0.90–1.30)	
Adventist	1.09 (0.85–1.40)	
Muslim	1.20 (0.68–2.10)	
Others	1.20 (0.67–1.81)	
Wealth index		**< 0.001**
Poorest	1	
Poorer	**0.49 (0.40–0.60)**	
Middle	**0.33 (0.26–0.43)**	
Richer	**0.22 (0.17–0.28)**	
Richest	**0.09 (0.06–0.13)**	
Residence		**< 0.001**
Rural		
Urban	**1.74 (1.29–2.35)**	
Region		**< 0.001**
South	1	
North	**0.63 (0.43–0.92)**	
East	**1.72 (1.28–2.30)**	
West	**1.40 (1.05–1.87)**	
Kigali	**3.35 (2.35–4.79)**	
Household size		**0.005**
6 and above	1	
Less than 6	**0.78 (0.65–0.93)**	
Sex of household head		**0.019**
Female	1	
Male	**0.78 (0.63–0.96)**	

*CI* Confidence interval, *N/A* Not applicable since only married women were selected for the domestic violence module, *RDHS* Rwanda demographic health survey

Bold values indicate significant, ** = significant at 0.05

## Discussion

In the present study, we used cross-sectional data from the nationally representative 2020 Rwanda demographic health survey to look into the relationship between health insurance utilization and women’s empowerment measures, as well as other socio-demographic variables in Rwanda. To our knowledge, this is the first study to evaluate the association between health insurance and women empowerment in Rwanda. Results indicated that the majority of Rwandan women (over 80%) reported owning health insurance, a higher prevalence than in most other study populations in East Africa [32, 42] and other countries [33, 35]. Mutual/community group insurance was the most widespread type of insurance, which is used by more than three-quarters of Rwandan women (77.2%). Such a high adherence to this type of insurance could be explained by the Rwandan government's early 2000s efforts to increase insurance coverage for vulnerable groups by supporting start-up initiatives, which resulted in the establishment of over 100 mutual or community health insurances. This campaign, which was followed by the passage of a law on mutual health insurance (law no. 65/2007) in 2008 requiring all Rwandans to participate in one or more types of health insurance schemes, resulted in a rapid surge in membership subscription [43, 44]. Private health insurance, as our statistics reveal, is the least popular option, with less than 1% of women opting for it. Notably, among the women’s empowerment indicators taken into account, decision-making and sexual empowerment had the highest proportion among Rwandan women, with 89% having medium to high decision-making power and 64.3% reporting high sexual empowerment.

Moreover, regarding the association with health insurance usage, three of the four indicators of empowerment—exposure to the media, decision-making, and economic empowerment had significant but negative associations. Women’s empowerment also includes their ability to make important decisions in their households. Exposure to useful and accurate information is critical for making informed decisions, especially when it comes to big decisions like selecting a health insurance policy. Paradoxically and in agreement with previous studies in similar settings [33, 36], our findings show that exposure to the media was linked to a lower likelihood of purchasing health insurance. One plausible explanation for this finding is that the media fails to effectively transmit health-related information and knowledge, or that the content is inadequate for the target demographic. As a result, as previously noted in other studies, there is an urgent need to examine and enhance existing communication methods by incorporating not only key stakeholders but also consumers in the design and implementation of health insurance communication programs [45]. Moreover, as our results indicate, a high proportion of Rwandan women are young, with over two-thirds (67.2%) falling into the 15–34 years age bracket. Globally, the use of the internet and social media has risen in recent years, particularly among the youth. According to a recent report, 31.4% of Rwandans now have access to the internet [46], as a corollary, social media outlets could be another effective tool to explore for spreading awareness by sharing transformative messages, leading to significant increases in health insurance enrollment. Actually, evidence from studies conducted in Ghana [47] and the United States [48] asserts that using social media to spread health insurance information has a high potential for positive effects in terms of health insurance uptake.

Our research also found that Rwandan women with high decision-making power and those with high economic empowerment were less likely to utilize health insurance compared to their respective deprived counterparts. This could be explained by previous research findings that showed women with more autonomy in healthcare decision-making being less likely to seek healthcare [49]. Empowered women, who are often also well-educated and come from wealthier backgrounds usually face fewer hurdles to accessing health care services, hinting that they could be able to finance out-of-pocket costs and thereby being less likely to need health insurance [50]. We also discovered that, contrary to earlier findings [34, 36, 37, 51], women with no education and those from the poorest households were also more likely to get health insurance compared to their respective privileged fellows. The aforementioned could therefore be traced to Rwanda's efforts to decentralize funding for healthcare delivery and health systems at the district level, ensuring that every community's needs are specifically addressed [52].

In comparison to women who lived in rural regions, those who lived in urban areas had a higher likelihood of having health insurance. The majority of insurance companies are located in urban centres, making it easier for urban residents to seek and obtain more direct and accurate information. This finding is in accordance with what has been found in earlier research [32, 33]. The region of residence was also a strong predictor of health insurance ownership. Women in the Northern region, in particular, were less likely than women in the South to have health insurance. The difference in accessibility, which may increase the distance and difficulty in finding a suitable provider who has the health insurance scheme in which individuals might want to enrol, could be a plausible explanation for this geographic disparity in health insurance coverage. This, therefore, highlights the need to continue expanding healthcare and transportation infrastructure to improve geographical accessibility to health facilities and services throughout the country.

Moreover, household size had a significant role in the decision to enrol on a health insurance scheme. Compared to smaller families, large families were more likely to enrol in a health insurance plan, which could be because large families would like to limit the high cost of healthcare since the out-of-pocket expenses for such a large number of household members would be significant and difficult to cover. This agrees with studies from Ethiopia [53, 54] but contrasts with studies from Ghana that instead found larger households being frequently uninsured or only partially insured [55, 56].

Health insurance enrollment was also influenced by the gender of the household head. When compared to male-headed households, being from a female-headed household was associated with a higher likelihood of having health insurance; a finding similar to Kimani et al. in Kenya [36] and Kibret et al. in Ethiopia [53]. This could be related to the fact that single-parenting women are frequently burdened with multiple household responsibilities, leaving them with little opportunity to explore formal, high-paying employment [57]. With no one else to support the family, financial difficulties and a low socioeconomic position are common, and thus consequently, more likely to consider joining a health insurance plan- as a fallback strategy/plan.

### Study strength and limitations

This is the first nationwide analysis that explores women empowerment and its association with health insurance utilisation and therefore, it can be used as a yardstick and motivation for further studies on the same topic in Rwanda and other countries. Additionally, we used the most recent nationally-representative dataset, making our findings generalizable to all women in Rwanda. However, our study has some limitations worth acknowledging, including, the cross-sectional design which doesn’t allow the establishment of causal relationships, but rather only associations. The use of self-reported answers and the possibility of giving false answers due to social desirability risks recall and information bias. There was also a lack of data on other key determinants of health insurance utilisation such as a history of chronic medical conditions, mandatory insurance subscription, and possession of other non-health insurance (property insurance), among others, all of which could affect the uptake of health insurance services.

## Conclusions

The study assessed health insurance utilisation and its association with women empowerment, including other socio-demographics. Rwandan women had a high percentage of health insurance enrollments, and the Mutual/Community scheme had the most participants. However, empowerment indicators had a negative association with health insurance; having high media exposure, decision-making and economic empowerment reduced the likelihood of having a health insurance plan. Other socio-demographic factors influenced access and subscription to health insurance including education level, wealth index, residence and region, household size and sex of household head. Therefore, the results suggested context-specific evidence for consideration when rethinking policies to increase health insurance coverage in this study group. Nonetheless, improving and tailoring media material considering specific knowledge gaps, as well as addressing misinformation could make media a powerful tool in increasing health insurance coverage. Addressing regional imbalance by improving women’s access to health facilities/services is also key in increasing the number of Rwandan women who have health insurance. Moreover, further research in other settings is needed to have a thorough understanding of how women’s empowerment relates with health insurance coverage.

## Data Availability

The data set used is openly available upon permission from the MEASURE DHS website (URL: https://www.dhsprogram.com/data/available-datasets.cfm). However, authors are not authorized to share this data set with the public but anyone interested in the data set can seek it with written permission from the MEASURE DHS website (URL: https://www.dhsprogram.com/data/available-datasets.cfm).

## Abbreviations

EA: Enumeration area
AOR: Adjusted odds ratio
UHC: Universal health coverage
MMR: Maternal mortality ratio
SDGs: Sustainable development goals
CI: Confidence interval
COR: Crude odds ratio
DHS: Demographic health survey
RDHS: Rwanda demographic health survey
OR: Odds ratio
VIF: Variance inflation factor
WHO: World Health Organization
SPSS: Statistical package for social science
COVID-19: Corona virus disease-2019
